# c.194 A>C (Q65P) mutation in the *LMX1B* gene in patients with nail-patella syndrome associated with glaucoma

**Published:** 2011-07-16

**Authors:** Pablo Romero, Felipe Sanhueza, Pamela Lopez, Loreto Reyes, Luisa Herrera

**Affiliations:** 1 Departamento de Oftalmología, Hospital Clínico José Joaquín Aguirre, Universidad de Chile, Santiago, Chile; 2 Servicio de Oftalmología, Hospital del Salvador, Santiago, Chile; 3 Servicio de Urgencia, Clínica Río Blanco, Los Andes, Chile; 4 Programa de Genética Humana, ICBM, Facultad de Medicina, Universidad de Chile, Santiago, Chile; 5 Banco de Sangre. Complejo Hospitalario Dr. Sótero del Río, Santiago, Chile

## Abstract

**Purpose:**

To report the clinical, ophthalmic, extraophthalmic, and genetic characteristics of nail-patella syndrome (NPS) in a Chilean family and to investigate the expressivity of open angle glaucoma (OAG) and ocular hypertension (OHT) in the family members.

**Methods:**

Five family members affected with NPS and two unaffected members underwent a complete ophthalmologic examination, including computerized visual field, optical coherence tomography (OCT) of the optic disc and ultrasound pachymetry. Renal function was assessed by urinalysis and blood tests. Orthopedic evaluations were also performed, including radiological studies of the wrist, elbow and hip joints. Genomic DNA was extracted from peripheral leukocytes of the five affected and two unaffected family members. Exons 2–6 of the LIM homeobox transcription factor 1-beta (*LMX1B*) gene were screened for mutations by DNA sequencing of the proband. We also screened for mutations in exon 2 by polymerase chain reaction-restriction fragment length polymorphism (PCR-RFLP) of the other participants and 91 blood donors.

**Results:**

Five living family members from three generations were positively diagnosed with NPS, three of them with varying degrees of OAG and one with OHT. Retinal nerve fiber layer (RNFL) thickness measured by spectral domain OCT was below normal values in three individuals. All subjects evaluated had normal nephrologic function. Orthopedic, clinical, and radiological alterations were compatible with NPS. Screening for mutations in exons 2- 6 of *LMX1B* showed a heterozygous missense mutation c.194 A>C changing glutamine to proline within exon 2 in codon 65 (Q65P) of the coding sequence. This mutation was present in all NPS subjects and absent in the unaffected family members and in 91 Chilean blood donors.

**Conclusions:**

This is the first report of c.194 A>C mutation in *LMX1B* in a Chilean family with NPS and the second worldwide. The phenotype associated with this mutation is variable within the family, although we noted a close connection between the presence of the c.194 A>C mutation and the presence of OHT or OAG and probably also with an early onset of OHT in patients with NPS. All subjects older than 21 years had either OHT or OAG. We also suggest that the *LMX1B* mutation may be related to affective disorders.

## Introduction

The nail-patella syndrome (NPS, OMIM 161200), also known as hereditary osteo-onychodysplasia, is an autosomal dominant disorder characterized by dysplasia of the nails, patellar aplasia or hypoplasia, glomerulopathy, and glaucoma [1-3]. This rare pleiotropic disease occurs in about 1 in 50,000 live births [4].

NPS is caused by a loss of function mutation in the LIM homeobox transcription factor 1-beta (*LMX1B*) gene at chromosome 9q34 encoding a LIM (Lin-1, Isl-1 and Mec-3)-homeodomain transcription factor. *LMX1B* is required for normal embryo development, in particular for dorsal limb development, kidney morphogenesis, the development of the anterior segment of the eye, and of the dopaminergic and serotonergic neurons [5-8].

The *LMX1B gene* encompasses 82,100 base pairs (bp) and comprises eight exons. It encodes a highly conserved protein of 379 amino acids containing two cystine-rich zinc-binding motifs (LIM-A and LIM-B domains) at the NH_2_-terminus, a homeodomain in the middle, and a COOH-terminal glutamine- and serine-rich domain [9,10]. The homeodomain is involved in DNA binding, the LIM domains are thought to regulate DNA binding of the homeodomain by interacting with other nuclear proteins, and the COOH-terminal domain represents a transcriptional activation domain [10].

To date, The Human Gene Mutation Database at the Institute of Medical Genetics in Cardiff has described 157 different mutations of *LMX1B*, including missense/nonsense (88 entries), splice-site mutations (18 entries), small insertions (8), small deletions (29 entries), small indels (4), gross deletions (8), gross insertions (1), and complex rearrangements (1) [11]. These mutants are concentrated in LIM-A and LIM-B domains encoded by exons 2–4, and in the homeodomain encoded by exons 4–6. In Chile, there are no previous reports of mutations causing NPS.

No clear genotype–phenotype correlations among patients with *LMX1B* mutations have been established so far. In fact, specific mutations of *LMX1B* may lead to wide phenotype variability for skeletal abnormalities, presence and severity of nephropathy and ocular anomalies [12,13]. The ocular abnormalities most associated with NPS are ocular hypertension (OHT) and open angle glaucoma (OAG) [4,14-16]. Other ocular anomalies related to NPS are infrequent and include microcornea, sclerocornea, congenital cataract, abnormal iris processes, pigmentation of the inner margin of the iris, and congenital glaucoma [4,14-16].

In this article, we report the ophthalmic, extraophthalmic clinical characteristics and a genetic mutation in *LMX1B* in a Chilean family having NPS with glaucoma. The c.194 A>C mutation segregates with NPS and we propose that this mutation is associated with early onset of either OHT or OAG.

## Methods

### Participants

All examinations were performed according to the tenets of the Declaration of Helsinki and the present study was approved by the ethics committee of the Clinical Hospital of University of Chile, Santiago, Chile. Blood donors were recruited from the blood bank of Sotero del Rio Hospital of Santiago, Santiago, Chile. All subjects were informed about the study and signed a consent form.

One index case was identified during an ophthalmic examination in the Clinical Hospital of University of Chile. We recruited information on five consecutive generations of this Chilean family by interviewing the proband. After obtaining informed consent six family members, five affected with NPS and one unaffected, were enrolled in the study. A clinical geneticist, an orthopedist, and an ophthalmologist evaluated them. NPS diagnosis was based on dysplastic nails, absent or hypoplastic patellae, exostoses (“horns”) of the ilia, and dysplasia of the elbows.

### Ophthalmologic evaluations

All participants received a detailed clinical examination which included best corrected visual acuity (BCVA) according to the best line of Snellen charts. Biomicroscopy of the anterior segment and dilated fundus examination were performed. Special attention was paid to the aspect of the optic nerve head (ONH). ONH photography, intra-ocular pressure (IOP) and computerized visual fields (CVF) were performed in all subjects. ONH photography was done with Zeiss Digital Imaging System, FF 450 plus (Zeiss FF 450 plus; Carl Zeiss AG, Jena, Germany). To evaluate the posterior segment, all subjects received pupillary dilatation with 0.5% tropicamide. IOP was measured with a Perkins applanation tonometry (Perkins tonometry Clement-Clarke Inc., Columbus, OH), using the mean of three consecutive tonometry readings for each eye. Gonioscopy was performed with a Sussman indentation gonioscope (Volk Optical Inc., Mentor, OH); funduscopy for the observation of the ONH and peripapillary region under direct examination with a 78 D Volkmann (Volk Optical Inc., Mentor, OH) lens completed the slit-lamp exam. CVF was done with a Humphrey Visual Field analyzer II following a SITA Standard strategy; C 24–2 program (750; Humphrey Instruments, Inc., Dublin, CA). We used a size III stimulus and a white-on-white test.

Ocular hypertension was defined by an IOP **>**21 mmHg on two separate occasions and normal cup–disk ratio [17]. Glaucomatous optic disk alteration was defined by a cup–disk ratio of ≥0.7 or a difference of at least 0.2 between the eyes in the cup–disk ratio accompanied by notching of the rim and/or peripapillary hemorrhages of the optic rim [17].

Primary open-angle glaucoma (POAG) was defined by glaucomatous cupping of the optic disk, glaucomatous visual field defects, an IOP **>**21 mmHg on two separate occasions, and open anterior chamber angle [18,19].

Optical coherence tomography (OCT) was performed with OCT version 3 (Stratus OCT; Carl Zeiss Meditec Inc., Dublin, CA), which is a high-resolution cross-sectional imaging technique that allows in vivo measurements of the retinal nerve fiber layer (RNFL) [20]. Central corneal ultrasound pachymetry was performed under topical anesthesia with an Ocuscan RxP pachymeter (Alcon Laboratories Inc., Irvine, CA) that provides six central readings and calculates the mean value.

### Orthopedic evaluations

A general evaluation specifically of the hands and the musculoskeletal system was done. For assessment of patellar aplasia or hypoplasia in subjects, a radiographic series was performed on both knees of individuals, including an antero-posterior view, a true lateral view and an axial view. A lateral view of the elbows of subjects IV-2 and an antero-posterior view of the pelvis in all subjects were taken.

### Evaluation of renal function

The evaluation of renal function was performed in all subjects of the family. Blood pressures were measured in supine position by Dynamap (Criticon, Tampa, FL). The determination of protein and albumin excretion and microscopic examination of urine sediment were performed in a first morning urine specimen. Serum creatinine and albumin were measured by standard chemical procedures. Microalbuminuria was defined as an albumin–creatinine ratio (ACR) between 30 and 300 mg/g and macroalbuminuria as a ratio of 300 mg/g or greater. Proteinuria was defined as total urinary protein ≥0.1 g/l accompanied by a protein–creatinine ratio ≥0.2 g per 10 mM creatinine.

Hypertension was defined as systolic blood pressure ≥140 mmHg and/or diastolic blood pressure ≥90 mmHg [21]. Individuals using antihypertensive therapy were also considered as being hypertensive. Glomerular filtration rate (GFR) was estimated by endogenous creatinine clearance (CrC), calculated by the Cockcroft–Gault formula and adjusted for body surface area [22,23].

### Molecular genetic analysis

Peripheral blood (5 ml) was collected from 5 affected and 1 unaffected family members and genomic DNA was isolated. Briefly,  DNA was isolated from whole blood by a rapid non enzymatic method, involving salting out of the cellular proteins by dehydration and precipitation with saturated sodium chloride solution. DNA was finally precipitated with absolute ethanol and washed with ice-cold 70% ethanol [24]. Exons 2–6 of *LMX1B* were screened for mutations by DNA sequencing. Briefly, genomic DNA was amplified by PCR using the pair of primers 5′-CTG ACG GCC GGG CTT TC / AAG ACG CGC AGC TCT CGG AA-3′ to amplify exon 2, 5′-GGC AGG AGT GGC CTC TG / TCC AGG ACA CCC CAG CAA C-3′ to amplify exon 3, and 5′-GTG TCA ACA GAG GGG ACA GG / TCT GCC CCA GCT CAC CCT G-3′ to amplify exons 4 to 6 in one fragment.

PCR amplification was performed as follows: an initial step of denaturation at 95 °C for 5 min was followed by 35 cycles of 1 min each step, including denaturation at 95 °C, annealing at either 58 °C (exon 2), 62 °C (exon 3), or 65 °C (exons 4–6) and elongation at 72 °C, and a final extension at 72 °C for 5 min. PCR products were resolved by electrophoresis in 3.% agarose gels, stained with ethidium bromide, and visualized under ultraviolet light. PCR products were sent for sequencing to Macrogen® DNA Sequencing Service (Macrogen, Seoul, Korea).

To analyze the same mutation in 91 anonymus subjects, we designed a rapid detection method based on polymerase chain reaction-restriction fragment length polymorphism analysis (PCR-RFLP). Briefly, genomic DNA was amplified using the primers 5′-TGC AGT GCG CgG CGT GTC gGC-3′ (forward) and 5′-CCT TGG AGC TGC CCA GCC CA-3′ (reverse). PCR amplification was performed as follows: an initial step of denaturation at 95 °C for 5 min was followed by 35 cycles of 1 min each step including denaturation at 95 °C, annealing at 68 °C and elongation at 72 °C, and a final extension at 72 °C for 5 min. Then the 212 bp PCR product was digested with HaeIII restriction enzyme for 3 h at 37 °C. Fragments were resolved by electrophoresis in a 3.5% agarose gel, stained with ethidium bromide and visualized under ultraviolet light. After digestion, the “A” allele consisted of three fragments of 152, 55, and 5 base pairs (bp) and the “C” allele consisted of four fragments of 132, 55, 20, and 5 bp.

## Results

**Figure 1:**
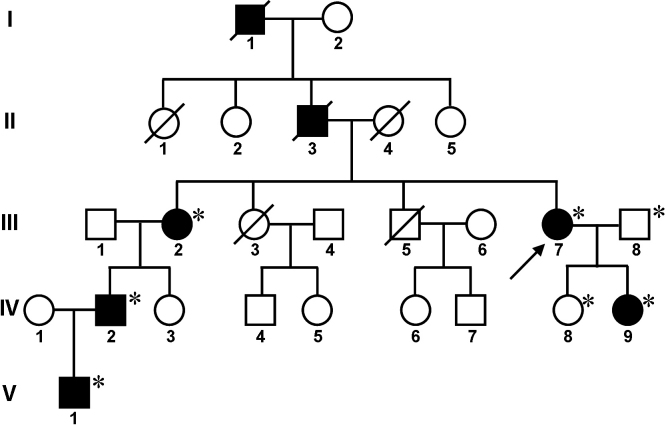
Pedigrees of the family affected by NPS. Pedigree showing five consecutive generations of affected members. Autosomal dominant transmission of the disease is evident. The arrow at the lower left of the symbol indicates the proband, squares indicate males and circles indicate females, open and filled symbols indicate unaffected and affected individuals respectively. Asterisks indicate members of the family who underwent clinical examination and molecular analyses.

The proband was a 51-year-old woman (III-7) who complained of difficulty in reading. The pedigree of the family was delineated (Figure 1). From an interview of subjects III-2 and III-7, NPS could be traced back over two more generations inferred according to the phenotype described (hypoplastic nails). That information revealed two dead subjects with history of NPS, subjects I-1 and II-3 (Figure 1). The paternal grandfather of the propositus (I-1) died from a cerebrovascular attack at age 75 years, and her father (II-3) had colon cancer, had depression and committed suicide at age 72. The propositus and six relatives were examined clinically. Five members of the family were affected by NPS, two males and three females, none of whom had previous diagnosis of NPS, and two individuals were unaffected (Figure 1). The presence of five continuous generations with NPS in the pedigree indicates an autosomal dominant transmission. The clinical features of the affected subjects are summarized in Table 1. Subject II-1 had a history of depression and committed suicide, patients III-2 and III-7 have a history of major depressive disorder and subject IV-2 has alcohol and drug addiction problems (Table 1).

**Table 1:** Clinical features in the patients affected with NPS.

**Patient**	**Gender**	**Age (Y)**	**Nail dysplasia**	**Hypoplastic patella**	**Nephropathy**	**OAG or OHT**	**Other manifestations**
III-2	F	63	Yes	Yes	No	Yes	Depressive disorder
III-7	F	51	Yes	Yes	No	Yes	Exostosis and depressive disorder
IV-2	M	42	Yes	Yes	No	Yes	drug and alcohol addiction
IV-9	F	21	Yes	Yes	No	Yes	
V-1	M	19	Yes	Yes	No	No	Congenital pulmonary stenosis

The phenotypic features of five patients affected by NPS are shown. The following abbreviations were used male (M), female (F), years old (Y), open angle glaucoma (OAG), and ocular hypertension (OHT).

### Ophthalmologic findings

The Ophthalmologic findings are summarized in Table 2. Gonioscopy showed open angles in all five patients.

**Table 2:** Ophthalmic features in the NPS patients.

**Patient**	**BCVA**	**IOP (mm Hg)**	**cup disc radio**	**Neuroretinal rim**	**Humphrey visual field testing**	**OCT**	**Diagnosis**
III-2	OD:0.2	OD: 14–15	OD: 0.9	OD: Complete loss of NRR	OD: Central island of vision	OD: Loss of RNFL	OAG
	OS:NPL	OS: 18–21	OS: 1.0	OS: Complete loss of NRR	OS: -	OI: Loss of RNFL	
III-7	OD:1.0	OD: 18–23	OD: 0.4	OD: Normal	OD: Nasal step in the right eye	OD: Loss of RNFL	OAG
	OS:1.0	OS: 20–24	OS: 0.4	OS: Loss in inferior temporal NRR	OS: Superior arcuate scotoma	OI: Loss of RNFL	
IV-2	OD:1.0	OD: 26–38	OD: 0.3	OD: Loss in superior and inferior NRR	OD: Superior and inferior nasal depression	OD: Loss of RNFL	OAG
	OS:1.0	OS: 27–35	OS: 0.3	OS: Loss in inferior temporal NRR	OS: Superior nasal step	OI: Loss of RNFL	
IV-9	OD:1.0	OD: 27–30	OD: 0.3	OD: Normal	OD: Normal	OD: Normal	OHT
	OS:1.0	OS: 25–31	OS: 0.3	OS: Normal	OS: Normal	OS: Normal	
V-1	OD:1.0	OD: 16–19	OD: 0.3	OD: Normal	OD: Normal	OD: Normal	Normal
	OS:1.0	OS: 15–18	OS: 0.3	OS: Normal	OS: Normal	OS: Normal	

The following abbreviations were used: (BCVA) Best-corrected visual acuity, (IOP) Intra-ocular pression, (NPL) no perception of light, (NRR) Neuroretinal rim, (OD) Right Eye, (OS) Left Eye, (OCT) Optical coherence tomography, (OAG) open angle glaucoma, and (OHT) ocular hypertension.

#### Case III-7

**Figure 2:**
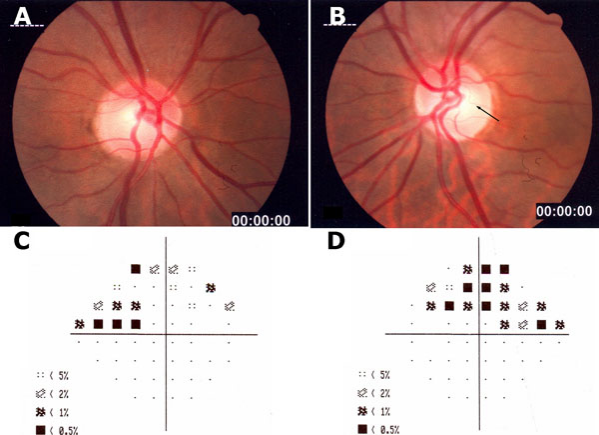
Photographs of the optic discs of glaucomatous eyes and visual fields of the proband. The optic disc photograph shows that the neuroretinal rim was lost slightly in her inferior temporal optic disc sector. The left eye shows greater loss of neuroretinal rim (**B**, arrow) than the right eye (**A**). The visual field shows a nasal step in the right eye (**C**) and a superior arcuate scotoma in the left eye (**D**).

The proband was a 51 year old woman. Her BCVA were 20/20 in both eyes, with normal color vision. Slit-lamp biomicroscopy of the anterior segment was normal and gonioscopy showed an open angle in both eyes. IOP varied between 18 and 23 mmHg in the right eye and between 20 and 24 mmHg in left eye, without treatment. The cup-disc ratio was 0.4 in both eyes. The neuroretinal rim was lost slightly in her inferior temporal optic disc sector in the left eye and retinas appeared normal in both eyes (Figure 2A,B). Central corneal ultrasound pachymetry was 559 μm and 553 μm in the right and left eyes, respectively. Humphrey visual field testing showed a nasal step in the right eye and a superior arcuate scotoma in the left eye (Figure 2C,D). Both optic nerve damage and visual field loss indicate the presence of glaucoma. Stratus OCT showed a loss of the infero-temporal RNFL in both eyes (not shown).

#### Case III-2

**Figure 3:**
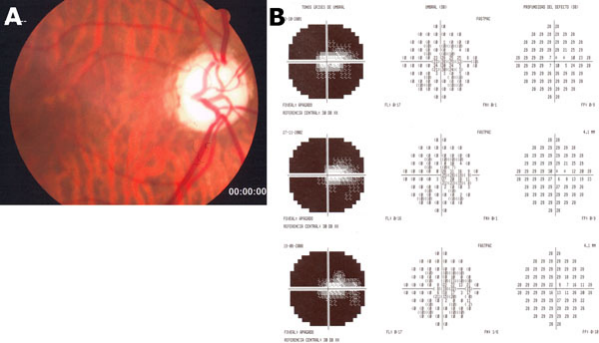
Photographs of the optic discs and visual fields of subject III-2. This fundus photography of the right eye shows a cup-disc ratio of 0.9 of the optic disc (**A**). Humphrey visual field testing of the right eye revealed a central island of vision (**B**). The visual field has remained stable during a period of eight years.

The proband's sister, a 63-year-old female, had been treated for glaucoma for over ten years. She was blind in her left eye due to glaucoma and had a history of trabeculectomy in both eyes at age 53 years. She used timolol 0,5% eyedrops twice a day in her right eye. BCVA was 0.2 in her right eye and she had no perception of light in her left eye. IOP varied between 14 and 15 mm/Hg in the right eye and between 18 and 21 mm/Hg in the left eye. Gonioscopy showed an open angle in both eyes. Funduscopic examination revealed loss of neuroretinal rim and very deep excavation in both optic discs with a cup-disc ratio of 0.9 in her right eye (Figure 3A) and total excavation in her left eye (not shown). Central corneal ultrasound pachymetry was 535 μm and 527 μm in the right and left eyes, respectively. Humphrey visual field testing of the right eye revealed a central island of vision which represents the terminal stage of the visual field. No significant changes in the visual field were observed during eight years (Figure 3B). Stratus OCT showed significant thinning of the nerve fiber layer in all quadrants in both eyes (not shown).

#### Case IV-2

**Figure 4:**
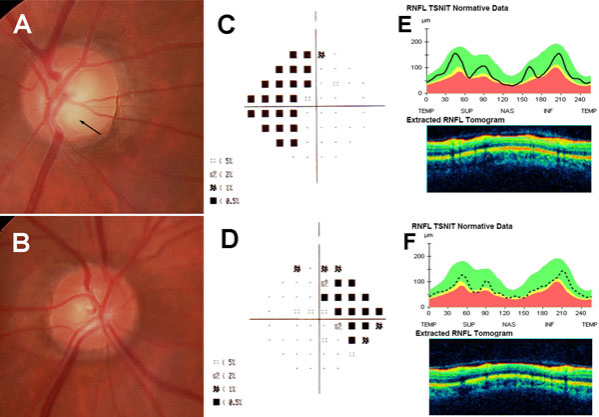
Photographs of the optic discs, visual fields and OCT of the subject IV-2. Optic disc photographs show a cup-to-disc ratio of 0.5 in the right eye and 0.2 in the left eye (**A** and **B**). The arrow shows the slight loss of the neuroretinal rim in the inferior temporal optic disc sector of the right eye (**A**). The visual field showed a superior and inferior nasal depression in the right eye (**C**) and a superior nasal step in the left eye (**D**). The continuous black line represents the thickness of the RNFL of the right eye (**E**) and the dashed line the left eye (**F**). The bottom of the OCT image shows the RNFL thickness and superior image-related patient data (black line) with normative data obtained from a normal population (**E** and **F**). In both eyes, the black line is located within the green band, which is considered normal, but in some areas runs into the yellow band which is considered suspicious for glaucoma.

The proband’s nephew was a 42-year-old male. His visual acuity was 20/20 in both eyes, with normal color vision. Slit-lamp biomicroscopy of the anterior segment was normal and gonioscopy showed an open angle in both eyes. His cup-disc ratio was 0.5 in the right eye and 0.2 in the left eye and the neuroretinal rim was lost slightly in his inferior temporal optic disc sector (Figure 4A, arrow) in the right eye. IOP without treatment varied between 26 and 38 mmHg in the right eye and between 27 and 35 mmHg in the left eye. Central corneal ultrasound pachymetry was 567 μm and 563 μm in the right and left eyes, respectively. The visual field showed a superior and inferior nasal depression in the right eye and a superior nasal step in the left eye (Figure 4C,D, respectively). Stratus OCT showed a loss of RNFL in both eyes (Figure 4 E,F).

#### Case IV-9

The proband’s daughter was a 21-year-old female. Her visual acuity was 20/20 in both eyes, with normal color vision. Slit-lamp biomicroscopy of the anterior segment was normal and gonioscopy showed an open angle in both eyes. Her optic discs and retinas appeared normal (not shown). IOP varied between 27 and 30 mmHg in the right eye and between 25 and 31 mmHg in the left eye. Central corneal ultrasound pachymetry was 552 μm and 561 μm in the right and left eyes, respectively. Humphrey visual field testing was normal in both eyes.

#### Case V-1

Case V-1 was a 19-year-old male. His visual acuity was 20/20 in both eyes, with normal color vision. Slit-lamp biomicroscopy of the anterior segment was normal and gonioscopy showed an open angle in both eyes. His optic discs and retinas appeared normal (not shown). IOP varied between 16 and 19 mmHg in the right eye and between 15 and 18 mmHg in the left eye. Central corneal ultrasound pachymetry was 541 μm and 548 μm in the right and left eyes, respectively. Humphrey visual field testing was normal in both eyes.

#### Cases IV-3 and IV-8

The healthy niece (IV-3) and daughter (IV-8) of the proband were completely normal. Visual acuity were 20/20 in both eyes, IOP were under 15 mmHg and slit-lamp biomicroscopy of the anterior segment and gonioscopy were normal.

### Orthopedic and nail alterations

All members of this family who were diagnosed with NPS had nail and orthopedic/radiological alterations.

#### Case III-2

**Figure 5:**
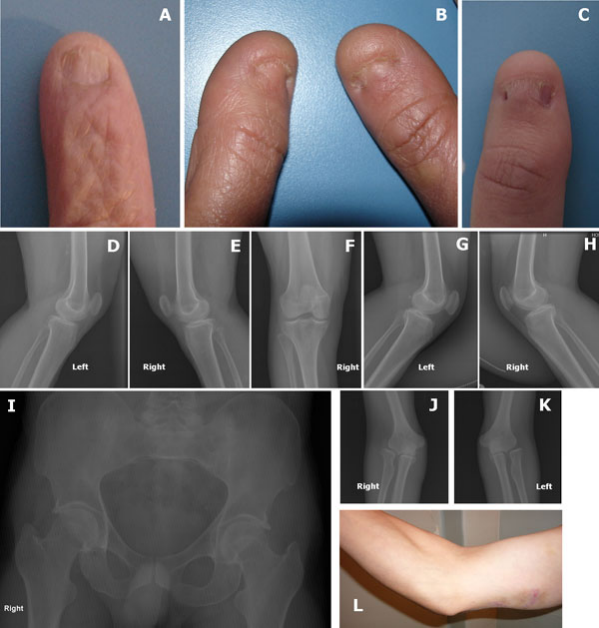
Photographs of the nails and radiographs of knees, hips and elbows of some of the subjects. Nail involvement in these cases includes dystrophic, thin, hypoplastic discolored nails with longitudinal ridges. Case III-2 shows decreased creases over the skin of distal interphalangeal joints, which is a sensitive sign of digital involvement in this patient (**A**). Thumbs are most severly affected in case III-7 (**B**), and IV-9 (**C**). The radiographs of knee involvement in Case III-2 shows bilateral involvement of the patella, with hypoplastic, higher than normal missplaced patella (**D**-**F**). A small 6 mm bone fragment can be observed on the lateral border of right patella (**E**). Knee radiographs of Case III-7 show slight bilateral patellar hypoplasia, mostly on the transverse diameter (**G** and **H**). Hip radiography of Case IV-2 shows loss of the normal concavity at the junction between the head and femoral neck bilaterally. The radiographs reveal an elbow involvement of subject IV-2 (**J** and **K**) and include a prominent medial epicondyle and hypoplasia of the capitellum on both sides (**J** and **K**). They also show a hypoplasia of the lateral epicondyle and capitellum with a slight deformity of the radial head (**J** and **K**). Underdeveloped triceps and prominent medial epicondyle are observed (**L**).

Nail alterations in this case included hypoplastic thin and discolored nails; with loss of creases in the skin overlying the distal interphalangeal joints (Figure 5A). The knee antero-posterior view showed a misplaced patella, in a higher than normal position, which can be observed in both knees (Figure 5D-F). The right patella also has a small bone fragment of 6mm on the lateral side (Figure 5E).

#### Case III-7

Nail alterations included small, hypoplastic and discolored nails (Figure 5B). There was slight bilateral patellar hypoplasia, mostly on the transverse diameter (Figure 5G,H). The patient had a history of dorsal exostoses surgery on both feet at age 42 years.

#### Case IV-2

Nail alterations in this case included dystrophic, thin and discolored nails with longitudinal ridges (not shown). The most severely affected nail was the thumb. The severity of alteration decreased progressively to the fifth digit (not shown). The hip radiograph showed loss of the normal concavity at the junction between the head and femoral neck bilaterally (Figure 5I). The antero-posterior X-rays of the elbows of this patient showed hypoplasia of the capitellum and of the lateral epicondyle and a prominent medial epicondyle on both sides (Figure 5J,K). Clinical examination also showed an underdeveloped triceps (Figure 5L).

#### Case IV-9

The nails in this case were dystrophic, discolored and partially separated in 2 halves by a longitudinal ridge of skin (Figure 5C).

#### Case V-I

The nails were dystrophic and had hypoplastic patellae in both knees (not shown).

#### Case IV-3 and IV-8

The healthy niece (IV-3) and daughter (IV-8) of the proband did not have orthopedic or nail involvement. Similarly, they do not have addiction or mood disorder history.

### Other clinical findings

All patients who underwent evaluation of renal function, urinalysis and blood tests exhibited normal values, and they had no clinical evidence of nephropathy. Cases III-2, III-7 and IV-9 had a history of urinary incontinence. Patient V-1 required intervention for congenital pulmonary valvular stenosis in the perinatal period. In all patients blood pressure was in normal range.

### Molecular genetic analyses

**Figure 6:**
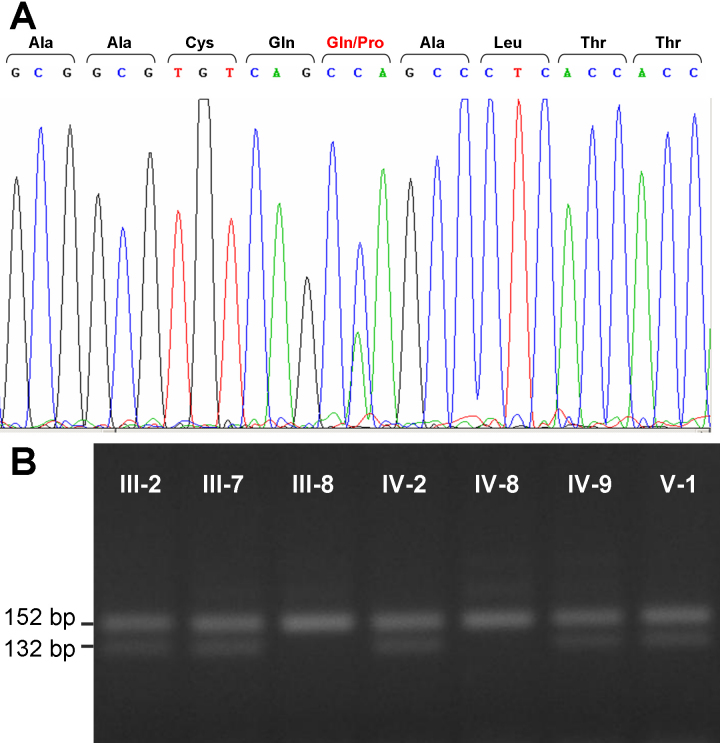
Direct sequencing of exon 2 of *LMX1B* in the proband (individual III-7) and PCR-RFLP analyses of the family. The DNA sequence around the codon of Glutamine 194 (CAA) of *LMX1B* is presented. The sequence shows a heterozygous, single-base A→C transversion at nucleotide 194 resulting in the replacement of glutamine (CAA) by proline (CCA; c.194 A>C) in codon 65 (Q65P). The amino acid sequence is indicated at the top of the figure (**A**). PCR-RFLP analyses of individuals III-2, III-7, III-8, IV-2, IV-8, IV-9, and V-1 were performed. Exon 2 was amplified from each available family member and digested with HaeIII. The products were analyzed by a 3.5% agarose gel electrophoresis (**B**).

Exons 2 to 6 of *LMX1B* of the proband were analyzed by sequencing. The sequence of exon two revealed that all affected individuals carry a heterozygous missense mutation c.194 A>C, changing codon 65 from CAA to CCA, which changes glutamine to proline (Q65P) in the *LMX1B* gene product (Figure 6A). Exon 2 of individuals III-2, III-7, IV-2, IV-9, and V-1 were further analyzed by DNA sequencing and PCR-RFLP. This mutation was present in all affected individuals and absent the two unaffected family member studied (Figure 6B). In the sequence analysis of codons 4 to 6 we identified two polymorphic sequences: rs13295990, a synonymous nucleotide variant in exon 4 at nucleotide position 657, and rs34682917, a C>T transition in intron 3. The minor alleles were present in both healthy and affected subjects (data not shown). The c.194 A>C mutation was screened by PCR-RFLP in 91 blood donors, although it was not detected.

## Discussion

In the present study we described the ophthalmic, orthopedic, and other phenotypic characteristics in a Chilean family with NPS. We also described the c.194 A>C mutation in *LMX1B*, causing the change of the amino acid glutamine to proline in position 65 (Gln65Pro). In the same family we found the single nucleotide polymorphisms (SNPs) rs34682917 in intron 3 and rs13295990 in exon 4. The minor alleles were present in both healthy and affected subjects with no association between any of the alleles and NPS (data not shown).

An ocular abnormality (OHT or OAG) was observed in 4 of the 5 patients with NPS. We observed one patient with a severe damage caused by glaucoma and most of the patients, who were not under treatment for ocular hypertension, had intraocular pressures over 25 mmHg, demonstrating the severity of the clinical manifestation. The absence of OHT or OAG in patient V-1, age 19 years, may be explained by his youth; that he has not yet developed the ocular manifestations.

The c.194 A>C mutation was present only in affected subjects (III-2, III-7, IV-2, IV-9, and V-1), and was neither observed in the two healthy subjects of the family (III-8 and IV-8) nor in 91 blood donors, supporting that the c.194 A>C mutation is not a common polymorphism. This mutation is located in exón 2 and was described once in an Australian family [2]. This mutation changes a glutamine located in the LIM-A domain, to proline. Proline is a unique amino acid, being its amino group part of the cyclical ring of atoms. Consequently, proline acts as a structural disruptor of proteins secondary structure such as alpha helices and beta sheets. The position involved in this change is placed close to one of the cysteines believed to be involved in a LIM-A domain zinc finger. An inclusion of proline near this cysteine likely alters the tertiary structure of the zinc finger, impairing the normal function of the LIM-A domain. Since LIM domains are involved in protein–protein interactions, the disruption of LIM-A domain zinc finger may alters the interaction with other nuclear proteins affecting the transcriptional activity regulated by LMX1B.

It has been described that 9.6% of subjects with NPS had glaucoma and an additional 7.2% had ocular hypertension (OHT); that is 16.8% of NPS patients developed either OHT or glaucoma [4]. The same data reviewed by Sweeney et al. [4] indicated that the mean age at diagnosis for glaucoma or OHT was 48 (range 23–78) years. Ophthalmic examinations and medical history of the five affected individuals evaluated in this study indicate that the c.194 A>C mutation in four out of five cases of NPS was associated with either OHT or OAG. Even more, 100% of those older than 21 years had either OHT or OAG. This suggests that this mutation might be a predictive factor for either OHT or glaucoma; and probably also for an early onset of these conditions. This is also supported by the data by Mimiwati et al. [2]. They described a family with 8 subjects with NPS and carrying the same c.194 A>C mutation [2], one female aged 54 years had neither OHT nor OAG; one 52-year old male had POAG diagnosed when he was 33 years old; one subject of 33 years old have OHT diagnosed when he was 24 years old, two subjects of 32 and 24 years old had IOP of 21 mmHg, and one subject of 10 years old have NPS and normal IOP. Two individuals of 50 and 17 years old respectively did not have glaucoma and there was no information about the IOP in the article [2]. Integrating their results with ours, at least 8 out of 11 subjects over 21 years old developed either OAG or IOP of 21 mmHg or higher, and one subject over 21 years old (54 year old) did not [2]. Two individuals under twenty years old, aged 10 [2], and 19 years (this study) had not developed OHT or OAG. Therefore, this data indicates that the c.194 A>C mutation might be predictive of OHT or OAG and also of an early onset of OHT.

On the other hand, extraocular symptoms were diverse in different subjects, including nail and orthopedic involvements. This variability might well be ascribed to epistatic effects, such as interactions with other developmental genes, especially considering the properties of *LMX1B*, whose LIM domain favors protein–protein interaction.

*LMX1B* is required for normal embryo development, including the development of dopaminergic and serotonergic neurons [5-8]. Moreover, serotonergic and dopaminergic neurotransmission systems have been associated with human behavior, including the pathophysiology of affective disorders, anxiety disorders, eating disorders and substance use disorders [25,26]. Along with this, depressive disorders are commonly associated with suicide attempts and completed suicide cases [27]. Based on this information, we suggest that the depressive history of subjects III-2 and III-7, the suicide committed by subject II-3, and the addictions of subject IV-2 may be related with a deficiency in the serotonergic and/or dopaminergic systems. As far as we know, there are no other nail patella cases associated with addictions or suicide previously, although NPS has been associated with Major Depressive Disorder (MDD) and Attention Deficit Hyperactivity Disorder (ADHD) symptoms [28].

This study highlights the need for further research into the function of LMX1B and its epistatic effects which develop a variety of phenotypes. We are especially interested in its role in the dopaminergic and serotonergic systems, along with its role in pathogenesis of glaucoma.
